# Integrated Tools for American Cutaneous Leishmaniasis Surveillance and Control: Intervention in an Endemic Area in Rio de Janeiro, RJ, Brazil

**DOI:** 10.1155/2012/568312

**Published:** 2012-09-04

**Authors:** Cheryl Gouveia, Rosely Magalhães de Oliveira, Adriana Zwetsch, Daniel Motta-Silva, Bruno Moreira Carvalho, Antônio Ferreira de Santana, Elizabeth Ferreira Rangel

**Affiliations:** ^1^Departamento de Endemias Samuel Pessoa, Escola Nacional de Saúde Pública Sérgio Arouca, Fundação Oswaldo Cruz, Rua Leopoldo Bulhões 1480, Manguinhos, 21041-210 Rio de Janeiro, RJ, Brazil; ^2^Laboratório de Transmissores de Leishmanioses, Instituto Oswaldo Cruz, Fundação Oswaldo Cruz. Avenue Brasil 4365, Manguinhos, 21040-360 Rio de Janeiro, RJ, Brazil

## Abstract

American cutaneous leishmaniasis (ACL) is a focal disease whose surveillance and control require complex actions. The present study aimed to apply integrated tools related to entomological surveillance, environmental management, and health education practices in an ACL-endemic area in Rio de Janeiro city, RJ, Brazil. The distribution of the disease, the particular characteristics of the localities, and entomological data were used as additional information about ACL determinants. Environmental management actions were evaluated after health education practices. The frequency of ACL vectors *Lutzomyia (N.) intermedia* and *L. migonei* inside and outside houses varied according to environment characteristics, probably influenced by the way of life of the popular groups. In this kind of situation environmental management and community mobilization become essential, as they help both specialists and residents create strategies that can interfere in the dynamics of vector's population and the contact between man and vectors.

## 1. Introduction 

American cutaneous leishmaniasis (ACL) is among the six most important infectious diseases and the 15 most neglected diseases of the world [1]. It presents a diversity of transmission cycles that involve different species of parasites, vectors, and hosts in restricted ecological niches [2]. Thus, the indication of control measures must consider the entomological and epidemiological characteristics of each locality.

According to Sabroza et al. [3], for each disease and particular situation there are environmental and behavioral factors related to the production of endemic or epidemic processes. To explain these factors, Sabroza et al. [3, page 216] used the concept of conditions receptivity, defined as the *“set of environmental, social and behavioral characteristics that allow the reproduction of the parasites and its maintenance in the communities*.*”*

The city of Rio de Janeiro presents many areas where these conditions are met, mainly because of human occupation of hillsides, which modifies the landscape and favors the installation of ACL transmission cycles. The number of cases in the city has been increasing since the 1980s, with the west zone presenting the highest indices, more specifically the region of Jacarepaguá [4]. Most of the cases in the study area (Campus FIOCRUZ da Mata Atlântica - CFMA, Rio de Janeiro) are related to the occupation of hillsides in Maciço da Pedra Branca, an Atlantic Forest area.

The present study aimed to discuss an experience on ACL surveillance based on integrated tools related to entomological surveillance, environmental management, and health education practices. At the same time, evidence is given for understanding the process whereby ACL develops at local level, identifying the social and biological determinants of the disease.

## 2. Materials and Methods

Quantitative and qualitative data produced under different perspectives, defined by Oliveira and Valla [5] as “construction of shared knowledge”, were used to describe the determinants of ACL at the endemic area.

### 2.1. Study Area: Campus FIOCRUZ da Mata Atlântica (CFMA)

CFMA is located in Jacarepaguá (west zone of Rio de Janeiro/RJ, Brazil) and was created in 2003, representing 65% of the former Colônia Juliano Moreira (CJM) (a psychiatric institution created in 1924 and extinct in the 1980s) and 50% of the Pedra Branca State Park (environmental preservation area) [6]. 

The campus is located next to the geographic center of the city and to the main highway that connects the north and south zones of Rio de Janeiro city. The limits of the campus are Pico da Pedra Branca at north and west, and urban sites at east [6]. 

CFMA is a region of Atlantic Forest and the areas above the altitude of 100 m are characterized as permanent preservation areas. However, the areas under that altitude present human-modified ecosystems [6]. 

The residential zone occupies 45% of the area of CFMA and comprehends five communities: Caminho da Cachoeira, Faixa Azul, Fincão, Sampaio Corrêa, and Viana do Castelo [6]. 

### 2.2. Characterization of the Distribution of ACL in the Study Area

Human cases registered by the Municipal Department of Health of Rio de Janeiro from 2001 to 2005 were used in this study. A database was constructed; the cases were added according to the patient's address and then analyzed.

The frequency of cases and the incidence of the disease were calculated using the population estimated by IBGE (Brazilian Institute of Geography and Statistics) in the demographic census of 2000 and the population of the localities estimated by FIOCRUZ in 2004 [6]. 

The analysis of the occurrence of ACL was based on the frequency of cases and the incidence of the disease, as well as on two variables; sex and age of the stricken inhabitants.

### 2.3. Description of the Determinants Associated with the Particular Characteristics of Each Locality

The first characterization of the localities was based on information provided by previous studies and research reports. Primary data were obtained from a systematic observation of the localities and from interviews with the inhabitants.

In each locality the systematic observation was carried out with a person who has lived there for many years. The following aspects have been observed: structure of the houses and their proximity to the forest, predominant use of the soil, garbage accumulation, vegetation cover, types of plantations, presence of banana trees and other fruitful trees, wild and domestic animals, and animal shelters.

The semistructured interviews were done between January and March of 2008 with privileged witnesses (Ethical Committee-CEP n. 62/07, approved on December 03, 2007) and investigated the inhabitants' knowledge on the occurrence of the disease in the campus. In all interviews a person who has lived in the locality for many years was the first informer. The choice of the other informers was made based on the indication by the oldest inhabitant and the time of residence in the locality. The aim was to interview inhabitants of all the localities, but the number of interviews was not established *a priori*; they were interrupted when the information became repetitive. Thus, 25 interviews were conducted: 8 in Caminho da Cachoeira, 5 in Fincão and Faixa Azul, 4 in Sampaio Corrêa, and 3 in Viana do Castelo. 

### 2.4. Description of Vector Ecology

The entomological study was developed in the community of Caminho da Cachoeira, which presented the highest number of cases of ACL amongst the localities of the campus.

This study followed the recommendations of the Brazilian Health Ministry [1], which include the following: taxonomy of sand fly fauna and identification of potential vectors of ACL; monthly frequency of vectors inside and outside houses, as well as within the limits of forests; seasonality of vectors; evaluation of vector behavior associated with its natural habitat. 

Sand fly captures were conducted from August 2006 to October 2009 in three houses, in different collection sites: domestic, peridomestic, and forest limit areas. Monthly captures were carried through three consecutive nights with HP light traps [7], similar to CDC light traps. On each house, three light traps were installed (one per site per night) from 4 PM to 8 AM. The terminology of the morphological characters and the identification of species followed the criteria indicated by Young & Duncan [8]. 

The description of the environmental characteristics of the collection sites of each house is given on Table 1. The possible influence of rainfall and temperature on vectors abundance was tested by linear regression, using the software SYSTAT 11. 

### 2.5. Health Education Practices

Considering the community's perception of ACL, workshops were conducted so that they could learn about the disease, its transmission, treatment, and control, following the methodology proposed by Gouveia [9]. The workshops were carried out during 2006.

### 2.6. Environmental Management

Systematic observations were conducted after the health education practices in order to register alterations in the local landscape and environmental. The impacts of environmental management on the sand fly fauna were analyzed by sand fly captures in the same house, using entomological indicators.

The environmental management actions spontaneously carried out by residents of the community were particularly related to those that would make the environment unsuitable for the breeding of immature stages and maintenance of adult sand flies.

## 3. Results

### 3.1. Distribution of ACL in the Study Area

From 2001 to 2005, 26 cases were registered in CFMA, corresponding to 8% of the cases in Rio de Janeiro in the same period.

Amongst the localities of CFMA, the community of Caminho da Cachoeira concentrated 65% of the cases, followed by Fincão with 27% and Viana do Castelo and Faixa Azul, both with 4% of the cases. The community of Sampaio Corrêa did not register any cases. As the frequency of cases, the disease's incidence was slightly higher in Caminho da Cachoeira (49.4 cases/1000 inhab.) followed by Fincão (48.6 cases/1000 inhab.). 

There are no records of the mucocutaneous form in the localities. ACL occurs equally in both genders, and the most affected age group is between 0 and 10 years (34%). 

The heterogeneous spatial pattern of ACL in Rio de Janeiro can also be observed in the communities of CFMA. Analyses from the cases database identified a trend to group the cases in specific points of the localities, disclosing an aggregation of familiar cases. 

### 3.2. Determinants Associated with the Particular Characteristics of the Localities

Although in general they have the same history of occupation, the communities of CFMA are heterogeneous in some aspects. Those who occupied the area first are more consolidated and have better social indicators, whereas the others have quite precarious conditions. Even internally, the localities have particular features that have justified their division in sectors, with the exception of Sampaio Corrêa and Viana do Castelo (Table 2).

### 3.3. Vector Ecology

More than 21,600 sand flies were captured and identified, including twelve species: *Brumptomyia brumpti, B. nitzulescui, Lutzomyia lutziana, L. edwardsi, L. hirsuta, L. (Micropygomyia) schreiberi, L. sordelii, L. pelloni, L. quinquefer, L. (Pintomyia) fischeri, L. (Nyssomyia) intermedia, and L. migonei*, the last two being regarded as vectors of ACL in Rio de Janeiro [10, 11]. 

A predominance of *L. (N.) intermedia* (96%) over *L. migonei* (3.2%) and other sand fly species (0.8%) was observed. Moreover, the density of both vectors is heterogeneous in the house and even in the collection sites (Table 1). 

**Figure 1:**
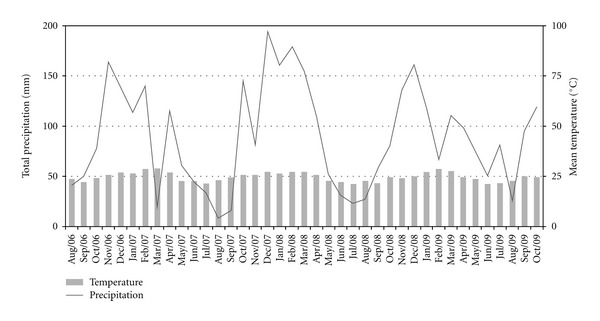
Monthly means of temperature (°C) and precipitation (mm) registered by INMET, in the west zone of Rio de Janeiro city, Southeast Brazil, August 2006 to October 2009.

Monthly total precipitation and mean temperature are shown on Figure 1. The linear regression analyses did not indicated any significant correlation between vectors abundance, rainfall, and temperature (*P* > 0.1).

**Figure 2:**
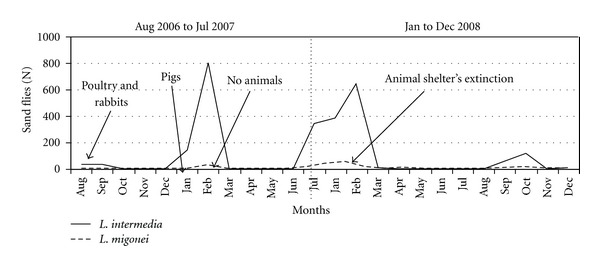
Environmental modifications and monthly capture of *L.* (N.) *intermedia* and* L. migonei* in the peridomicile of house 2, August 2006 to July 2007 and January to December 2008.

**Figure 3:**
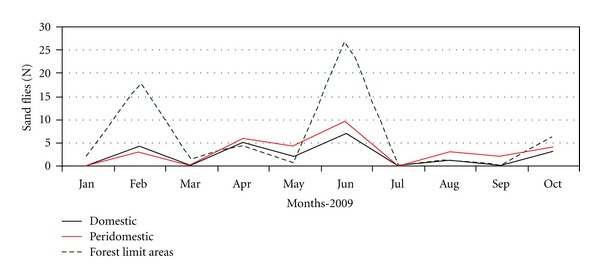
Monthly capture of sand flies vectors in domestic, peridomestic and forest limit areas of house 2, January to October 2009.

**Figure 4:**
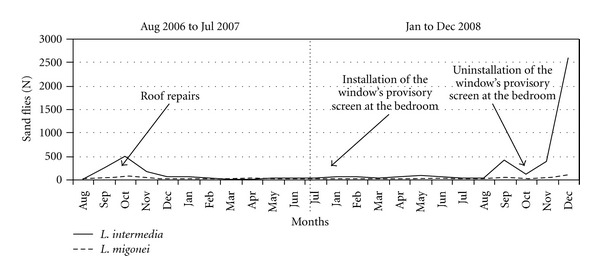
Environmental modifications and monthly capture of *L.* (N.) *intermedia* and* L. migonei* in the domicile of house 3, August 2006 to July 2007 and January to December 2008.

**Figure 5:**
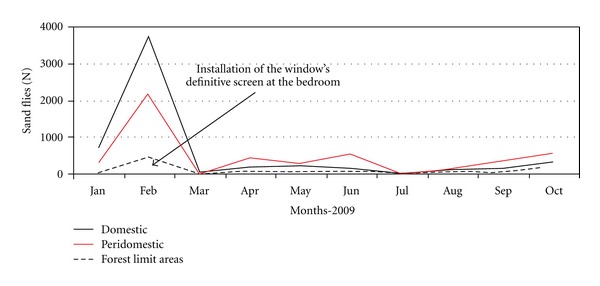
Environmental modifications and monthly capture of sand flies vectors in domestic, peridomestic, and forest limit areas of house 3, January to October 2009.

The captures showed that *L. (N.) intermedia* and *L. migonei* were present on every month of collection, but it was observed that the frequency of both species did not remain constant throughout the study period (Figures 2–5). 

### 3.4. Environmental Management

Some environmental management actions were developed by the inhabitants after the health education practices based on a previous study about their social perception of ACL. These actions resulted in environmental modifications that influenced the frequency and habits of vectors.

The number of sand fly specimens captured at house 1 was not expressive, and no vector species was collected during the study period at any collection site.

House 2: the modifications occurred in house 2 were related to the presence or absence of animal shelters in the peridomicile. From August to December of 2006, when few chickens and rabbits inhabited shelters, the peridomicile was responsible for 42% of *L. (N.) intermedia* and *L. migonei* collected in house 2 (Figure 2), with the predominance of the first one. When pigs were introduced in the shelter, from January to February of 2007, the frequency of these species in this site was 61%. The animals were removed in March and by the three next months those frequency was lesser than 10%. Pigs were reintroduced in the shelter between June and July of the same year and the frequency of *L. (N.) intermedia* and *L. migonei* was about 26% (Figure 2). This shelter was extinct in February of 2008, thus their capture in the peridomestic environment was drastically reduced (Figure 2). After that, during 2009, the frequency of both vectors at the forest limit areas was increased and the intradomicile had lesser density of sand flies (Figure 3). Figure 2 shows that *L. (N.) intermedia* and *L. migonei* frequencies were influenced by environmental management at this house.

House 3: The modifications in house 3 occurred after six months of sand fly captures and were related to structural improvements made in the house. The frequency of *L. (N.) intermedia* collected in the intradomicile from August 2006 to January 2007 was 71%. After the space between the roof and the walls was blocked, from February to July 2007, that frequency was reduced to 53% (Figure 4). The installation of a window's provisory screen in one bedroom of the house in January 2008 reduced the domestic density of *L. (N.) intermedia* (34.9%). The screen was removed in November of the same year and a new increase in the density of this vector was registered (Figure 4). A definitive screen was installed at the same place in February 2009 and the domestic density of both vectors was reduced. After that, the peridomicile (which contains a hen house and kennels) became the collection site with the highest sand fly frequency (42.4%) (Figure 5). The frequency of *L. (N.) intermedia* and *L. migonei* at house 3 also seems to have being influenced by the environmental management at this house.

## 4. Discussion and Conclusions

### 4.1. Determinant Factors and ACL Distribution

The current epidemiological pattern of ACL in Rio de Janeiro diverges from that observed in the beginning of the 20th century. Nowadays it is related to the population dynamics, the occupation of hillsides, and urban agglomerations around secondary or residual forests [1, 2, 11–21].

Studies developed in the city are intended to understand the occurrence of the disease based on the distribution of cases, the environmental characteristics, and the vector's behavior. However, considering the ACL epidemiology, it is important to associate these data with the history of the space occupation process.

Unlike other localities where ACL occurs, CFMA presents a singular history; it originated from the old Colônia Juliano Moreira, a psychiatric institute that promoted the occupation of its area by employees and patients' relatives in order to socially integrate the patients. It is interesting to observe that the organization of health services attracted groups for an area without proper infrastructure, which generated other public health problems.

This attraction reproduced the inequality in society, so employees occupied the most urbanized portion of the area, whereas the patients' relatives were established in the most distant areas.

This dynamics resulted in different receptivity conditions and vulnerability to ACL. This vulnerability is related to bigger difficulties in anticipating, controlling, and recovering from the impacts of different health risks [22].

In the period of study, ACL mainly occurred in Caminho da Cachoeira and Fincão, which presents an epidemiological pattern that has already been pointed out by other authors [12, 18, 23]. This pattern consists in the occurrence of the disease in diverse ages. Still in accordance with these authors [12, 18, 23], the main occupation of the inhabitants is not related to activities that involve the forest, but with formal and informal commerce and services, except for Fincão, where most people work on agriculture. The occurrence of the disease at ages between 0 and 10 years discloses a trend to the peridomestic and domestic transmission, as pointed out by Sabroza [12] in the 1980s.

Analyzing the particular characteristics of the localities, the existence of a peridomestic, and domestic transmission cycle becomes more evident. The spatial characteristics of localities with human cases are related to large amount of vegetation around residences, proximity of houses to the forest, frequent presence of wild animals (opossums, rodents, and primates) in the peridomicile, presence of domestic animals (cats, horses, and dogs), breeding of birds and swine, precarious basic sanitation, and lack of access to essential services.

The systematic observation within each locality sector allowed the observation of the existence of different risks of infection in the same locality, as pointed out in previous a study [12]. Inside the localities, the sectors that present more ACL cases reproduce the characteristics listed previously.

### 4.2. Vector's Ecology and Environmental Management

The entomological study added to the study of the particularities allowed an extended view of the dynamics of ACL transmission in CFMA. The presence of the vector was noticed on houses 2 and 3, possibly related to the particular characteristics already cited, such as the large amount of vegetation around residences, the proximity of houses to the forest, the frequent presence of wild and domestic animals in the peridomicile, the breeding of birds and swine, the precarious basic sanitation, and the lack of access to essential services.

The presence of animal shelters (mainly hen houses) next to the secondary forest, where ACL primary transmission cycle could be occurring, attracts sand flies to the peridomicile [24, 25]. 

The anthropophily of *L. (N.) intermedia* and its strong attraction for domestic animals (chickens, dogs, and equines) and for synanthropic rodents are factors that strengthen the hypothesis of the sand flies' attraction to the peridomestic environment [25, 26].

However the presence of sand flies in the peridomicile does not ensure the occurrence of ACL in a certain place; other important variables can influence it, such as sand fly frequency and infection index [27].

The captured vectors demonstrate great ability to adapt to anthropic environments, since they were captured in association with man and domestic animals [11, 28]. Thus, the presence of *L. (N.) intermedia* and *L. migonei* and their high abundance at the intradomestic (house 3) or peridomestic (house 2) environment can be associated with animal shelters and it is a common finding in studies conducted elsewhere [11, 16, 25, 29, 30].

Previous studies about seasonality indicate an irregular behavior of *L. (N.) intermedia* [31], with presence throughout the year and a higher frequency in cold months [11]. However, a higher frequency of the vector in hot months of the year was observed (January and February). *L. migonei* is known for not occurring during every month of the year, being absent in cold and dry months [31, 32]. However, this species was captured throughout the year, with lesser frequency in hot months (December and January).

Similar to the study of Teodoro et al. [25], the changes observed in the vector's behavior associated with the natural and anthropic habitat show that the monthly frequency of vector species is probably not influenced by meteorological conditions.

The changes observed in the sand fly frequencies at the peridomiciliar and intradomiciliar area after some measures were taken indicate that these dynamics can be influenced by the characteristics of the environment where people live. The reduction in the number of sand flies collected after environmental management was also noted by Teodoro et al. [25] at the peridomiciliar area of an endemic locality.

However, this environment is modified by man and its characteristics are related to the behavior and practices of its inhabitants.

Considering the particular characteristics of the localities studied, all of them have conditions for occurrence and maintenance of the ACL transmission cycle; however, the conditional variable of this dynamics is closely related to the way of life present in each locality. The ACL transmission dynamics varies even inside the same community, presenting different entomological indicators according to each way of life. It is possible to observe that the different risks of ACL transmission are probably associated with the way of life of a determined population, which influences the installation and maintenance of the disease's receptivity conditions.

The health education practices gave the inhabitants important insights on the vectors, their habits, and habitats, as well as on the transmission of the disease, resulting in environmental management actions which occurred naturally. It is important to highlight the initiative of residents (motivated to control the disease), which had an impact on the dynamics of the population of sand fly vectors.

Health education and environmental management, both of them recommended by the Brazilian Health Ministry, seem to be essential for entomological surveillance and prevention of the disease, as they reduce the presence and frequency of the vector in the peridomicile and intradomicile.

After the alterations conducted in the houses, only two ACL cases were registered in Caminho da Cachoeira community until today. It reinforces the necessity to think about environmental management actions capable of interfere on the process of ACL transmission without interfering on the way of life of the endemic localities.

According to WHO [33], one recommendation concerning leishmaniases control is to adopt an integrated vector management approach. In this sense, the understanding of local epidemiology, sand fly vectors, and their ecology is essential [25]. The success of these actions will obviously depend on social mobilization, government interventions, and assistance provided by health professionals [34].

## Figures and Tables

**Table 1:** Environmental characteristics of collection sites and frequency of sand fly vector species (Caminho da Cachoeira-Campus FIOCRUZ da Mata Atlântica, Jacarepaguá, Rio de Janeiro/RJ), August 2006 to October 2009.

House	Collection site	Characteristics	Frequency (%)*
1	Domestic	Brickwork residences, with spaces between roof and walls and no plaster or flagstone. Windows and doors are protected with glass, fabric, or plastic. Trap installed in the bedroom.	0.20
	Peridomestic	Dirt floor with grass and fruitful trees, such as banana trees. Absence of domestic animals.	0.25
	Forest limit areas	Distance of 20 meters from the house.	0.30

2	Domestic	Brickwork residences, with spaces between roof and walls and no plaster or flagstone. Windows and doors are not fully protected. Trap installed in the bedroom.	0.40
	Peridomestic	Dirt floor with abundant vegetation and fruitful trees, mostly banana trees. Presence of an animal shelter used to poultries, rabbits, and pigs. Garbage accumulation.	14.00
	Forest limit areas	Distance of 30 meters from the house.	4.40

3	Domestic	Brickwork residences, with spaces between roof and walls and no plaster or flagstone. Windows and doors are protected with glass. There are five dog shelters around the house. Trap installed into the living room, used as dormitory at night.	34.00
	Peridomestic	Dirt floor with grass and fruitful trees. Presence of poultries, dogs, and pigs. Garbage accumulation. Presence of banana plantation.	35.50
	Forest limit areas	Distance of 15 meters from the house.	11.00

*Total frequency of vector species per collection site and house.

**Table 2:** Synthesis of environmental characteristics of the localities in Campus FIOCRUZ da Mata Atlântica (Jacarepaguá, Rio de Janeiro/RJ).

Localities						Characteristics				Access to essential services
		ACL incidence	Configuration	Farming activity	Animals in peridomicile	Socioeconomic status	Basic sanitation		
							Light supply	water/sewer supply	Garbage collection
Caminho da Cachoeira	Sector I Sector II Sector III	10 cases/1000 inhab	84 residences and 334 inhabitants	No Yes Yes	Yes (domestic) Yes (wild and domestic) Yes (wild and domestic)	Mostly young. Medium-low education. Medium income. Trade and service sector.	Yes Yes Yes	Yes No No	Yes Yes No	Hard, because of the distance.

Faixa Azul	Sector I Sector II	4,5 cases/1000 inhab	12 residences and 44 inhabitants.	No No	Yes (domestic) Yes (domestic)	Mostly young. Low education. Low income. Informal trade.	Yes Yes	No Yes	Yes Yes	Hard, because of the distance.

Fincão	Sector I Sector II	9,7 cases/1000 inhab	40 residences and 144 inhabitants.	Yes Yes	Yes (wild and domestic) Yes (wild and domestic)	Few aged. Low education. Low income. Agricultural producers.	Yes Yes	No No	Yes Yes	Hard, because of the distance.

Sampaio Corrêa		0 cases/1000 inhab	43 residences and 134 inhabitants.	No	Yes (domestic)	Mostly adults. High education. High income. Trade and service sector.	Yes	Yes	Yes	Easy access.

Viana do Castelo		3,8 cases/1000 inhab	15 residences and 52 inhabitants.	No	Yes (domestic)	Mostly children. Medium education. Medium income. Trade and service sector.	Yes	Yes	Yes	Easy access.

## References

[1] Ministério da Saúde, Secretaria de Vigilância em Saúde (2007). *Manual De Vigilância Da Leishmaniose Tegumentar Americana*.

[2] Rangel EF *Estudo comparativo de três populações de Lutzomyia (Nyssomyia) whitmani (Antunes & Coutinho, 1939) (Diptera, Psychodidae, Phlebotominae) [Ph.D. thesis]*.

[3] Sabroza PC, Kawa H, Campos WSQ, Minayo MCS (1995). Doenças Transmissíveis ainda um desafio. *Organizadora. Os Muitos Brasis—SaúdE E População Na Década dE 80*.

[4] Kawa H, Sabroza PC (2002). Espacialização da leishmaniose tegumentar na cidade do Rio de Janeiro. *Cadernos de Saúde Pública*.

[5] Oliveira RM, Valla VV (2001). As condições e as experiências de vida de grupos populares no Rio de Janeiro: repensando a mobilização popular no controle do dengue. *Cadernos de Saúde Pública*.

[6] FIOCRUZ (2004). *Diagnóstico Do Setor 1 Da Colônia Juliano Moreira*.

[7] Pugedo H, Barata RA, França-Silva JC, Silva JC, Dias E (2005). HP: um modelo aprimorado de armadilha luminosa de sucção para a captura de pequenos insetos. *Revista da Sociedade Brasileira de Medicina Tropical*.

[8] Young DC, Duncan NA (1994). *Guide To Identification and Geographic Distribution of Lutzomyia Sandflies in Mexico, the West Indies, Central and South America (Diptera: Psychodidae)*.

[9] Gouveia C (2008). *Condições particulares de transmissão da Leishmaniose Tegumentar Americana em localidades do Campus FIOCRUZ da Mata Atlântica (Jacarepaguá, Rio de Janeiro/RJ) [Master’s dissertation]*.

[10] Rangel EF, Souza NA, Wermelinger ED, Azevedo AC, Barbosa AF, Andrade CA (1986). Phlebotomus of Vargem Grande, a focus of cutaneous leishmaniasis in the State of Rio de Janeiro. *Memorias do Instituto Oswaldo Cruz*.

[11] Rangel EF, Azevedo AC, Andrade CA, Souza NA, Wermelinger ED (1990). Studies on sandfly fauna (Diptera: Psychodidae) in a foci of cutaneous leishmaniasis in mesquita, Rio de Janeiro State, Brazil. *Memorias do Instituto Oswaldo Cruz*.

[12] Sabroza PC (1981). *O domicílio como fator de risco na leishmaniose tegumentar americana: estudo epidemiológico em Jacarepaguá, município do Rio de Janeiro [Master’s dissertation]*.

[13] Lainson R (1983). The American leishmaniases: some observations on their ecology and epidemiology. *Transactions of the Royal Society of Tropical Medicine and Hygiene*.

[14] Lainson R (1988). Ecological interactions in the transmission of the leishmaniases. *Philosophical transactions of the Royal Society of London. Series B: Biological sciences*.

[15] Toledo LM (1987). *Leishmaniose Tegumentar e Leishmaniose Visceral em Área Peri-Urbana no Município do Rio de Janeiro [Master’s dissertation]*.

[16] Rangel EF Transmission of American cutaneous leishmaniasis in peridomestic foci in Rio de Janeiro State and other similar situations compared to the classical epidemiology in Amazon region.

[17] Walsh JF, Molyneux DH, Birley MH (1993). Deforestation: effects on vector-borne disease. *Parasitology*.

[18] Kawa H (2003). *A produção do lugar de transmissão de leishmaniose tegumentar na cidade do Rio de Janeiro [Ph.D. thesis]*.

[19] Costa SM (2005). *Estudos de algumas populações brasileiras de Lutzomyia (Nyssomyia) whitmani s. 1. (Diptera: Psychodidae: Phlebotominae), importante transmissor de agentes da leishmaniose tegumentar americana [Master’s dissertation]*.

[20] Meneses CRV, De Azevedo ACR, Da Costa SM, Costa WA, Rangel EF (2002). Ecology of American cutaneous leishmaniasis in the state of Rio de Janeiro, Brazil. *Journal of Vector Ecology*.

[21] Meneses CRV, Cupolillo E, Monteiro F, Rangel EF (2005). Micro-geographical variation among male populations of the sandfly, *Lutzomyia (Nyssomyia) intermedia*, from an endemic area of American cutaneous leishmaniasis in the state of Rio de Janeiro, Brazil. *Medical and Veterinary Entomology*.

[22] Blaikie P, Cannon T, Davis I, Wisner B (1996). *Vulnerabilidad: El Entorno Social, Político y Económico de Los Desastres*.

[23] Oliveira-Neto MP (1998). *Leishmaniose tegumentar no Estado do Rio de Janeiro: estudo de 648 casos observados no Hospital Evandro Chagas [PhD thesis]*.

[24] Alexander B, de Carvalho RL, McCallum H, Pereira MH (2002). Role of the domestic chicken (*Gallus gallus*) in the epidemiology of urban visceral leishmaniasis in Brazil. *Emerging Infectious Diseases*.

[25] Teodoro U, Thomaz-Soccol V, Kühl JB (2004). Reorganization and cleanness of peridomiciliar area to control sand flies (Diptera, Psychodidae, Phlebotominae) in south Brazil. *Brazilian Archives of Biology and Technology*.

[26] Afonso MM, Gomes AC, Meneses CR, Rangel EF (2005). Studies on the feeding habits of *Lutzomyia (N.) intermedia* (Diptera, Psychodidae), vector of cutaneous leishmaniasis in Brazil. *Cadernos de Saúde Pública*.

[27] Pita-Pereira D, Alves CR, Souza MB (2005). Identification of naturally infected *Lutzomyia intermedia* and *Lutzomyia migonei* with *Leishmania (Viannia) braziliensis* in Rio de Janeiro (Brazil) revealed by a PCR multiplex non-isotopic hybridisation assay. *Transactions of the Royal Society of Tropical Medicine and Hygiene*.

[28] Aguiar GM, Vilela ML, Lima RB (1987). Ecology of the sandflies of Itaguaí, an area of cutaneous leishmaniasis in the State of Rio de Janeiro. Food preferences (Diptera, Psychodidae, Phlebotominae). *Memorias do Instituto Oswaldo Cruz*.

[29] Rangel EF, de Souza NA, Wermelinger ED, Barbosa AF (1984). Natural infection of *Lutzomyia intermedia* Lutz & Neiva, 1912, in an endemic area of visceral leishmaniasis of Rio de Janeiro. *Memorias do Instituto Oswaldo Cruz*.

[30] Araújo-Filho NA (1978). *Epidemiologia da leishmaniose tegumentar na Ilha Grande [Master’s dissertation]*.

[31] Forattini OP (1973). *Entomologia Médica*.

[32] Barreto MP (1943). *Observações sobre a biologia em condições naturais, dos flebótomos do Estado de São Paulo (Diptera, Psychodidae) [Ph.D. thesis]*.

[33] World Health Organization (2010). Control of the leishmaniases: report of a meeting of the WHO expert committee on the control of leishmaniases.

[34] Warburg A, Faiman R (2011). Research priorities for the control of phlebotomine sand flies. *Journal of Vector Ecology*.

